# A Liquid Crystal-Modulated Metastructure Sensor for Biosensing

**DOI:** 10.3390/s23167122

**Published:** 2023-08-11

**Authors:** Siyuan Liao, Qi Chen, Haocheng Ma, Jingwei Huang, Junyang Sui, Haifeng Zhang

**Affiliations:** College of Electronic and Optical Engineering & College of Flexible Electronics (Future Technology), Nanjing University of Posts and Telecommunications, Nanjing 210023, China

**Keywords:** metamaterial, liquid sensing, narrowband absorber, tunable device

## Abstract

In this paper, a liquid crystal-modulated metastructure sensor (MS) is proposed that can detect the refractive index (RI) of a liquid and change the detection range under different applied voltages. The regulation of the detection range is based on the different bias states of the liquid crystal at different voltages. By changing the sample in the cavity that is to be detected, the overall electromagnetic characteristics of the device in the resonant state are modified, thus changing the position of the absorption peaks so that different RI correspond to different absorption peaks, and finally realizing the sensing detection. The refractive index unit is denoted as RIU. The range of the refractive index detection is 1.414–2.828 and 2.121–3.464, and the corresponding absorption peak variation range is 0.8485–1.028 THz and 0.7295–0.8328 THz, with a sensitivity of 123.8 GHz/RIU and 75.6 GHz/RIU, respectively. In addition, an approach to optimizing resonant absorption peaks is explored, which can suppress unwanted absorption generated during the design process by analyzing the energy distribution and directing the current flow on the substrate. Four variables that have a more obvious impact on performance are listed, and the selection and change trend of the numerical values are focused on, fully considering the errors that may be caused by manufacturing and actual use. At the same time, the incident angle and polarization angle are also included in the considered range, and the device shows good stability at these angles. Finally, the influence of the number of resonant rings on the sensing performance is also discussed, and its conclusion has guiding value for optimizing the sensing demand. This new liquid crystal-modulated MS has the advantages of a small size and high sensitivity and is expected to be used for bio-detection, sensing, and so on. All results in this work were obtained with the aid of simulations based on the finite element method.

## 1. Introduction

Metastructures, as artificially fabricated periodic topological subwavelength structures, have many excellent properties that are not available in artificial materials due to their specific three-dimensional special structures. They have gradually become a hot research topic across the globe. Metastructures can realize many extraordinary physical properties, such as a negative refractive index, an inverse Doppler effect, etc. Using the extraordinary physical properties of metastructures, sensors, filters, and couplers [1,2,3] can be designed, as well as novel functional devices that conventional materials cannot achieve, such as perfect absorbers [4,5,6], waveguides [7,8,9], photonic crystals [10], and metalenses [11,12]. One of the important research directions for metastructures in the current research is to transform them from simple planar structures to three-dimensional spatial structures. Such metastructures focus on the coupling relationship between spatial structures and can often achieve more flexible multifunctional design, thus becoming a hot field of multifunctional and integrated devices.

In the design of superstructures, tunable materials are often introduced, and liquid crystals are one of the important materials for voltage tuning. A liquid crystal (LC) is a material existing in a state of matter between the liquid and crystalline states. As an emerging tunable dielectric, LC has a tunable dielectric constant that can be continuously adjusted by applying a bias to orient the LC molecules. Due to its unique characteristics, including a low insertion loss, a wide operating band, low cost, and stable electromagnetic properties, especially at higher frequencies, it has advantages that other materials do not have. The material properties of LCs have been widely used in microwave and millimeter wave bands and are gaining attention in the electromagnetic field. Electromagnetic devices, including metastructures, have made outstanding progress with the help of LCs [13,14,15,16,17].

A common approach in the design of metamaterial-based sensors is to sense with the help of narrow-band absorption produced by an absorber. A perfect absorber is an important application of metastructures. Since it was first proposed in 2008, it has attracted considerable interest owing to its attractive characteristics, which encompass nearly perfect absorption, a flexible design, and a thin thickness. The application of the absorber is divided into two directions: broadband absorption [18,19,20] and narrowband absorption [21,22,23]. Among them, since the absorption peak of narrow-band absorption is often generated by resonance, it is easy to relate the frequency of the absorption peak to the external environment. This natural advantage can be exploited in applications such as sensing and detection [24,25,26], as well as broadband absorption, because it has irreplaceable advantages over traditional materials in terms of thickness, weight, etc. Therefore, it has a wide range of applications in various applications, including electromagnetic shielding, electromagnetic pollution prevention, multiplexing technology, and so on.

With the rapid development of the sensor industry, sensor devices have become indispensable components in various fields, including information technology, biotechnology, agriculture, etc. Due to their advantages of real-time detection, no pollution of the detected samples, etc., electromagnetic sensors in the terahertz band are considered to be a type of detector device with broad prospects. With the help of transmission spectrum analysis, absorption spectrum analysis, the optical rotation effect, the Hall effect, and other principles, metastructure sensors can be used to detect various physical quantities, such as the dielectric constant, thickness, angle, etc., greatly enriching the realization methods and detection means of sensors. In 2023, Guo et al. fabricated a sensor that could measure the concentration of glucose solution using an open square resonant ring [27]. Chen et al. used the principle of electromagnetic resonance coupling to produce a biosensor capable of detecting low concentrations of cytomas [28]. In 2022, Ismail et al. achieved sensing detection against coronaviruses using narrow-band absorption [29].

Considering the rapid growth of the bio-industry, there is a growing requirement for sensing and detection. There are still many interesting materials in the field of sensing that are not commonly utilized. Therefore, it is necessary to continue to diversify the use of sensing materials and device design. In this paper, a metastructure sensor (MS) modulated by a LC is proposed, which can realize refractive index (RI) detection in the THz band, enriching the design of LC sensors. The performance of the MS is given in Table 1. The orientation of the long axis of the LC can be controlled by the applied voltage. The orientation of the initial state is in the horizontal direction, and the voltage is applied in the vertical direction. The long axis of the LC points in the vertical direction when the full-bias state is applied. In the initial state, the detection range of the refractive index is 1.414–2.828, the absorption peak shifts from 1.028 THz to 0.8485 THz, and the sensitivity is 123.8 GHz/RIU. In the full-bias state, the detection range is 2.121–3.464, and the absorption peak shifts from 0.8328 THz to 0.7295 THz, with a sensitivity of 75.6 GHz/RIU. In addition, in the design process, it often faces the problem of multiple resonant peaks, that is, there will be redundant small absorption peaks around the absorption peak, so that the frequency and RI of the absorption peak cannot meet the injective relationship. This problem is discussed in this paper with the help of an energy density diagram and current flow direction, and a scheme to suppress the appearance of redundant absorption peaks is proposed. In addition, the effects of the errors in each parameter on performance are explored in detail, taking into account the possible errors in the manufacturing and actual use of the MS. The number of resonant rings, angles of incidence, and polarization are also equally indispensable parts used to fully consider the possibility of optimizing the device. The proposed MS has the advantages of a small size, tunability, a large measurement range, and high accuracy, which can be used for biosensing and other applications.

## 2. Theory and Model

In this paper, a high-frequency simulator structure (ANSYS Electronics Desktop) was selected for the simulation, and the finite element method was used for the solution. The *x-y* plane was set to periodic boundary conditions, and the *z*-direction was set to open space.

**Figure 1:**
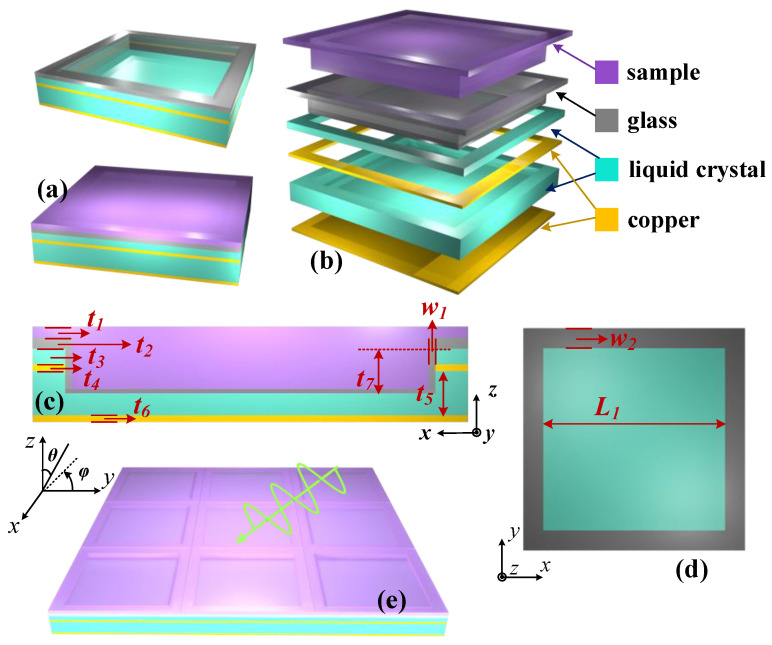
Schematic diagram of the MS structure: (**a**) unit of the sensor before and after filling with the liquid sample to be measured; (**b**) schematic diagram of the separated multi-layer structure; (**c**,**d**) size diagram of the unit, where *t*_7_ is the depth of the filled cavity and *w*_1_ is the thickness of the glass wall of the cavity; (**e**) schematic diagram of the array with incident electromagnetic waves (EMWs), with the incident angle *θ* and polarization angle *φ* indicated.

Figure 1 shows the structure of the MS, and the parameters of each dimension are given in Table 2. According to the structure diagram, the designed MS consists of a six-layer structure, in which the top layer is filled with the liquid sample to be measured, followed by the glass layer for confining the liquid crystal, the liquid crystal, the copper resonance ring in the liquid crystal, the liquid crystal, and the metal base plate, respectively. Among them, the resonant ring is in contact with the glass layer, thus creating a possibility for fabrication.

Among them, the conductivity of copper is *σ* = 5.8 × 10^7^ S/m [30]; the permittivity of glass is *ε* = 4 [31]; the long-axis RI of the liquid crystal is *n_e_* = 1.799; and the short-axis RI of the liquid crystal is *n_o_* = 1.513 [32]. In accordance with the axes defined in Figure 1, the initial orientation of the LC is defined in this paper to be towards the *y*-axis while pointing to the *z*-axis in the full-bias state. With such a definition, the RI matrix of the LC can be expressed by Equation (1), where ***n_i_*** is the initial state and ***n_F_*** is the full-bias state. For the RI of the intermediate state, it can be analyzed from an equivalent point of view, and for the incident EMW, the equivalent refractive index is given in Equation (2), where *β* represents the angle of rotation. In this paper, the default incident linear polarization wave is polarized in the *y*-direction.

(1) $$n_{i}=\left[\begin{array}{ccc} n_{0} & 0 & 0\\ 0 & n_{e} & 0\\ 0 & 0 & n_{0} \end{array}\right]\;n_{F}=\left[\begin{array}{ccc} n_{0} & 0 & 0\\ 0 & n_{0} & 0\\ 0 & 0 & n_{e} \end{array}\right]$$

(2) $$n_{eff}=\sqrt{\frac{1}{\frac{\cos^{2}\beta }{n_{e}^{2}}+\frac{\sin^{2}\beta }{n_{0}^{2}}}}$$

**Figure 2:**
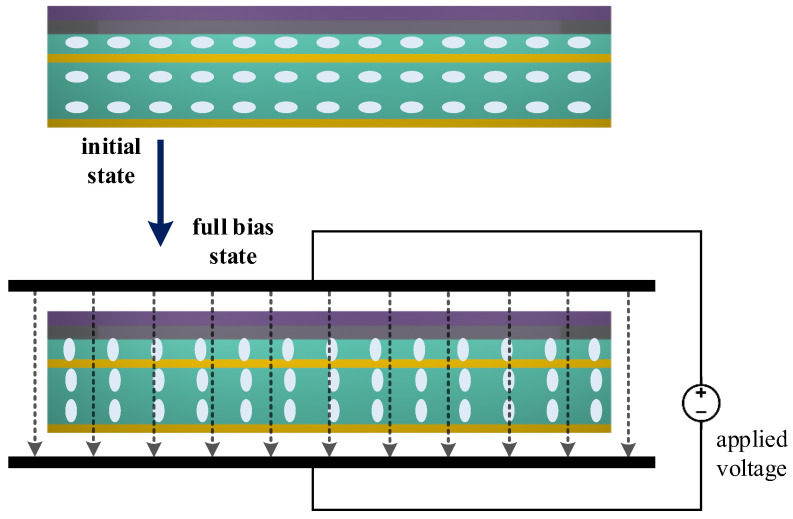
Schematic diagram of liquid crystal rotation with applied voltage.

As can be seen from Figure 2, the initial state of the liquid crystal is in the horizontal direction. After applying an external voltage, as the electric field strength gradually increases, the liquid crystal will gradually rotate and finally completely turn to the vertical direction. The specific full-bias voltage depends on the type of liquid crystal and the specific structure of the device. Based on this characteristic, for vertically incident EMW, the overall equivalent RI of the device will change under different voltages. In this paper, only the performance under zero voltage and full bias is discussed.

For an incident EMW, it can be orthogonally decomposed into components in the *x*- and *y*-directions. Thus, for an incident EMW in any direction, the reflected and transmitted EMWs can be written in the following form:

(3) $$E_{in}=\left[\begin{array}{c} E_{x}\\ E_{y} \end{array}\right]$$

(4) $$E_{ref}=\left[\begin{array}{cc} r_{xx} & r_{xy}\\ r_{yx} & r_{yy} \end{array}\right]E_{in}\;E_{tra}=\left[\begin{array}{cc} t_{xx} & t_{xy}\\ t_{yx} & t_{yy} \end{array}\right]E_{in}$$

(5) $$r_{xx}=\frac{E_{x}^{ref}}{E_{x}^{in}}\;r_{yx}=\frac{E_{y}^{ref}}{E_{x}^{in}}$$

(6) $$r_{yy}=\frac{E_{y}^{ref}}{E_{y}^{in}}\;r_{xy}=\frac{E_{x}^{ref}}{E_{y}^{in}}$$

(7) $$t_{xx}=\frac{E_{x}^{tra}}{E_{x}^{in}}\;t_{yx}=\frac{E_{y}^{tra}}{E_{x}^{in}}$$

In the above equation, ***E_in_*** denotes the incident EMW, ***E_ref_*** represents the reflected EMW, and ***E_tra_*** is the transmitted EMW. And *r_xy_* indicates the reflection coefficient of the EMW incident with *y*-polarization and reflected with *x*-polarization, namely, the cross-polarization reflection coefficient, and the other parameters are defined in the same way. Similarly, *r_xx_* represents the co-polarization reflection coefficient, *t_xy_* is the cross-polarization transmission coefficient, and *t_xx_* corresponds to the co-polarization transmission coefficient. Based on the above, when incidence is along the *y*-direction, the absorptivity *A* of EMW can be defined as

(8) $$A=1-{\left|r_{yy}\right|}^{2}-{\left|r_{xy}\right|}^{2}-{\left|t_{yy}\right|}^{2}-{\left|t_{xy}\right|}^{2}$$

Moreover, for the sensor, the Q-factor and figure of merit (FOM) are measures of sensitivity and detection accuracy. Defining the center frequency as *f*_0_, the half-wave width as FWHM, and the sensitivity as *S*, the Q and FOM are calculated as follows [33]:

(9) $$Q=\frac{f_{0}}{\mathrm{FWHM}}$$

(10) $$\mathrm{FOM}=\frac{S}{\mathrm{FWHM}}$$

**Figure 3:**
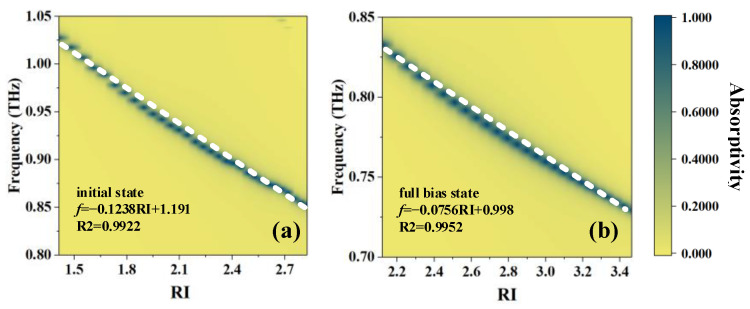
Schematic diagrams of the relationship between *f* and RI: (**a**) initial state and (**b**) full-bias state.

Based on the above equation, the position of absorption peaks can be calculated as shown in Figure 3, which gives the relationship between the frequency where the absorption peak is located and the RI. The frequency *f*–RI equation is obtained by curve fitting. In the two states, R^2^ equals 0.9922 and 0.9952, so the relationship between *f* and RI can be regarded as linear, which is convenient for the practical application of sensing.

(11) $$d=\sqrt{\frac{2}{\omega u\sigma }}$$

On the other hand, skin depth is also extremely important in the sensing process. The general calculation formula for skin depth is demonstrated in Equation (11), where *ω* is the angular frequency, *u* is the magnetic permeability, and *σ* represents the conductivity. Considering that the response frequency of the device is around 1 THz, the skin depth should be around 0.066 μm when the frequency equals 1 THz. The copper material used in the device has a thickness of more than 3 μm, so it can be considered that EMWs will not penetrate the device.

Based on the formula calculation, it can be seen that the Q-factor of the initial state varies between 130.54 and 178.73, and the main reason for the change is the shift in *f*_0_, while the width change of a single resonance peak is not very obvious. Therefore, in this case, the FOM is more suitable to show the performance within the working frequency band. The FOM of the initial state is 20 RIU^−1^. Similarly, the FOM of the full-bias state is around 13 RIU^−1^, and the Q-factor will be in the range of 122.39 to 162.2. The change in value is caused by the variation in the absorption frequency in combination with small differences in the width of the absorption peaks.

Based on the above performance, a possible application scenario is given. Since different components in the blood will affect the overall RI of the blood, the concentration of creatinine in the blood can be measured by the change in refractive index. Table 3 shows the relationship between concentration and RI [33]. Apparently, RI values corresponding to different concentrations are within the detection range.

## 3. Mechanism Analysis and Optimization

**Figure 4:**
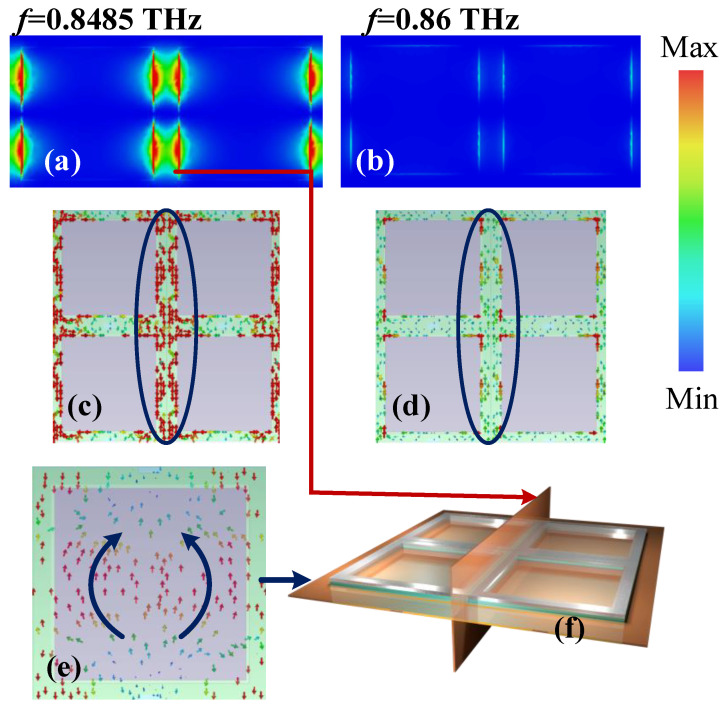
Energy density diagrams at (**a**) 0.8485 THz and (**b**) 0.86 THz when RI = 2.828. The surface current of the resonance ring at (**c**) 0.8485 THz and (**d**) 0.86 THz. (**e**) The surface current on substrate at 0.8485 THz. (**f**) The cross section diagram.

Figure 4 presents the energy distribution at the absorption peak (*f* = 0.8485 THz) and at *f* = 0.86 THz, where no absorption occurs when RI = 2.828. It can be seen that, compared with the frequency where no absorption occurs, a strong resonance appears clearly on the metal frame of the second layer between the two units at the absorption frequency. At the same time, the current on the copper ring (the third layer from the bottom up, as shown in Figure 1b) is also significantly enhanced, and the current on the bottom plate shows the current distribution of dipole resonance. Therefore, the absorption peak is caused by resonance. Therefore, the change in absorption frequency caused by different RI and LC orientations essentially changes the electromagnetic characteristics around the resonant part, thus changing the absorption frequency.

**Figure 5:**
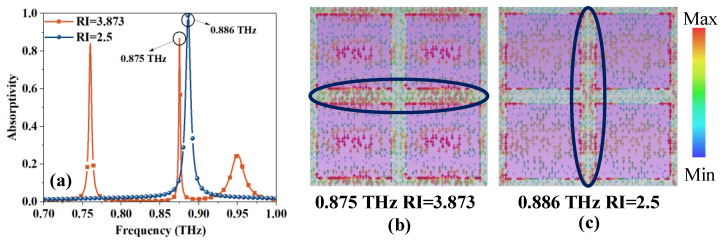
(**a**) Absorption peaks and excess absorption peaks at different RI values. Current diagram in different situations, with the strongest part of the current circled in the diagram: (**b**) 0.875 THz and RI = 3.873; and (**c**) 0.886 THz and RI = 2.5.

Although the absorption frequencies at different RI values present a good linear relationship, there are still excess absorption peaks at a few specific RI values. Although these excess absorption peaks do not reach 0.9 and their corresponding RI values are out of the measurement range, this brings risks for practical use due to the close proximity of the peaks and the overlap of the frequency bands. As demonstrated in Figure 5a, the absorption peaks that appear at the two values of RI in the initial state, 2.5 and 3.873, are quite close. The absorption peaks given in the figure are staggered, but since the blue absorption peak will move with the change in RI, a part of the blue peak must be located at 0.875 THz, corresponding to the RI value between about 0.25 and 2.65. Similarly, the position of the orange excess absorption peak will also change with the value of RI. According to the simulation results, the absorption peak that appears in the range of 3.6–4 for the RI will overlap with the detection band, which will cause errors in detection.

Figure 5b,c show the current maps in the two cases. It can be seen from the figure that the absorption peak at 0.875 THz is due to the vertical resonance, while the blue absorption peak used for detection is due to the horizontal inter-unit resonance. Therefore, the absorption can be weakened by cutting narrow slits on the bottom plate to block the current.

**Figure 6:**
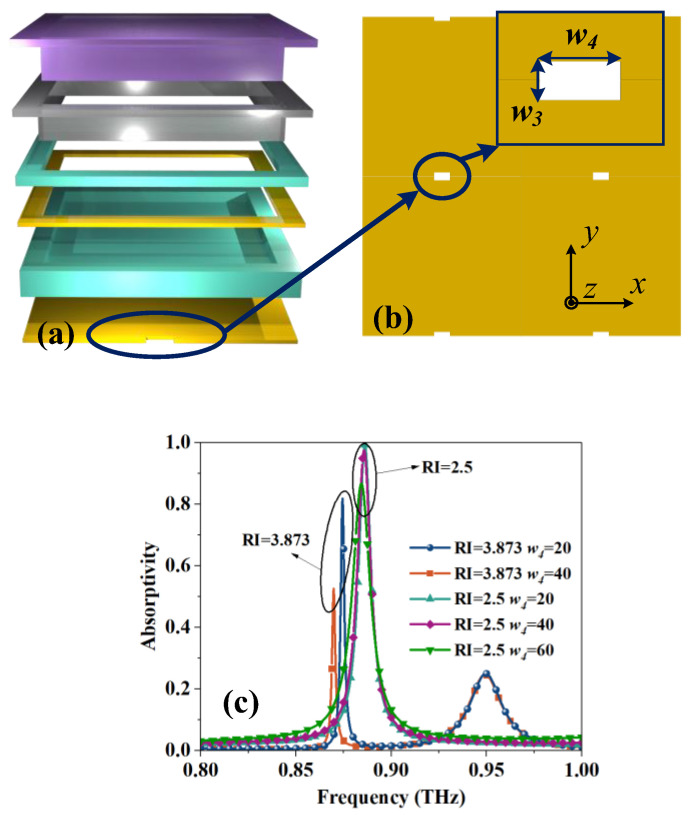
(**a**) A perspective view of the multi-layer structure. (**b**) Size diagram of the gap on the bottom plate, *w*_3_ = 10 μm. (**c**) Influence on absorption peak under different RI and *w*_4_.

The specific method is to etch slits on the bottom copper plate, as shown in Figure 6a,b. The two sets of curves with different *w*_4_ are shown in Figure 6c. As the value of *w*_4_ increases, the excess absorption is clearly suppressed. Until *w*_4_ = 40 μm is reached, the original absorption peak used for detection (RI = 2.5 group) did not show a significant decline. Only after continuing to increase to 60 μm does the absorptivity drop below 0.9; this is because the expansion of the slit will inevitably lead to an increase in transmittance and ultimately cause the absorptivity to decrease. Therefore, *w*_4_ values in the range of 20–40 μm are acceptable. Based on this operation, the absorption caused by excess resonance can be effectively suppressed to ensure the accuracy of sensing.

## 4. Results and Discussion

The main reason for the shift of the absorption peak is that the measured object changes the electromagnetic characteristics of the MS as a whole, so the change of the structural parameters should have a similar effect on the absorption peaks at each frequency point. Considering this, at the critical point of the detection range, the absorptivity will slightly decrease, and the linear relationship of the fitting will become slightly worse. Therefore, all the following results are given in the initial state, when RI = 2.65. The fitting results here have better linearity, and the change in a single absorption peak can represent the change trend of all absorption peaks within a certain range.

**Figure 7:**
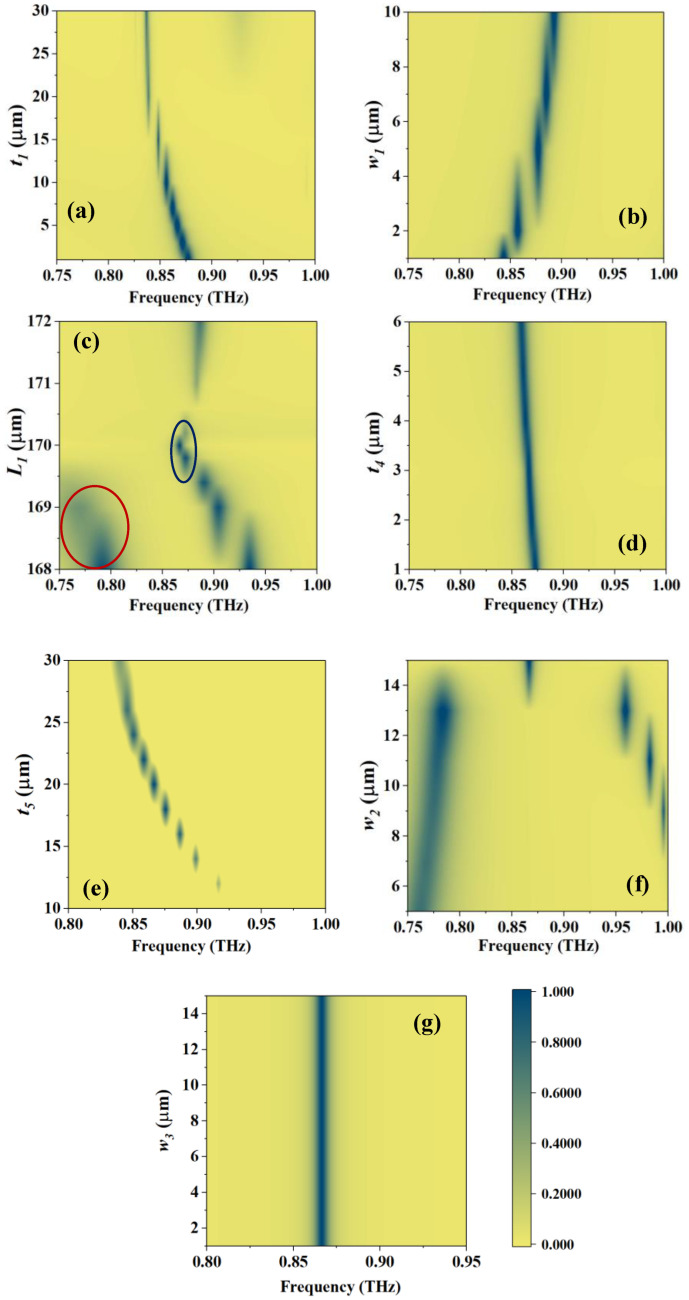
Effects of different parameters on the absorption peaks at RI = 2.65: (**a**) *t*_1_, (**b**) *w*_1_, (**c**) *L*_1_, red circles are additional absorption peaks that appear at low frequencies, and blue circles are optimal selection values, (**d**) *t*_4_, (**e**) *t*_5_, (**f**) *w*_2_, and (**g**) *w*_3_.

The filling thickness of the sample is one of the most important indicators in practical applications. Figure 7a shows the effect of thickness *t*_1_ on the absorption peak. It can be seen that in the region near 5 μm, the value of the absorptivity is relatively stable, and only the frequency shift is more obvious. This phenomenon can be intuitively understood as follows: when the thickness of the filler changes for a MS, its equivalent RI also changes. Therefore, such a property can also be applied to thickness detection and other aspects when the RI of the sample is known. When the thickness continues to increase, for EMWs, the influence of the MS gradually decreases, and the state becomes closer to EMWs directly incident to the sample interface; thus, the absorptivity decreases. According to the RI = 2.65 calculation, the incidence reflectivity is about 0.45, which means that the maximum absorptivity is 0.55. It can be seen that as the thickness increases, the absorptivity decreases and becomes closer to this value.

The errors in the manufacturing process should also be fully considered. There are some sensitive parameters, which are the thickness *w*_1_ of the glass wall, the side length *L*_1_ of the filled groove, the thickness of the copper ring *t*_4_, the distance from the substrate to the copper ring *t*_5_, and the width of the copper ring *w*_2_. The absorption peak is insensitive to other parameters, and at least no obvious difference is shown in a fairly large range of changes. 

As illustrated in Figure 7b, the absorptivity corresponding to different *w*_1_ is relatively stable, and the absorption peak gradually moves to a high frequency as the thickness increases. *L*_1_ is a very sensitive variable, and only when it is near 170 μm can a stable absorption peak be formed, as marked in the center of Figure 7c. When the value is greater than 170 μm, the absorption quickly decreases, completely disappears at 170.6 μm, and then gradually increases, but it is difficult to return to above 0.9 in a large range. When *L*_1_ is less than 170 μm, although it can maintain an absorptivity above 0.9 and is accompanied by some movement, it will excite an extra absorption peak in the low frequency range (circled in red), which overlaps with part of the detection range. Moreover, with the frequency shift, the Q-factor of the absorption peak also shows a more serious attenuation, which is not conducive to detection.

The influence of the thickness t4 of the copper ring on the absorption peak is shown in Figure 7d. From the offset of the absorption peak and the numerical value, it can be found that as the numerical value gradually increases, the absorption frequency point gradually shifts to a low frequency, and the absorption rate shows a trend of first increasing and then decreasing. Among them, *t*_4_ = 3 μm is the maximum point, and then the absorption rate gradually decreases. At *t*_4_ = 1 μm, although the absorption rate is not much different from that at 3 μm, its bandwidth is slightly increased, which will have a negative impact on the accuracy of sensing. Therefore, considering the situation comprehensively, choosing *t*_4_ = 3 μm is the most suitable.

For *t*_5_, the first thing to emphasize is that it corresponds to the distance between the copper ring and the bottom plate. The change in *t*_5_ only indicates the up and down movement of the copper ring, and the sum of the LC thicknesses of its upper and lower layers is constant. Therefore, considering the manufacturing possibility and the overall thickness of the LC, the range of the change in *t*_5_ is between 10 and 30 μm. Figure 7e shows the absorption peaks at different *t*_5_ values. It can be seen that as *t*_5_ increases, the absorption point first moves toward low frequency and stabilizes after increasing to 25 μm. On the other hand, *t*_5_ = 20 μm is the optimum point of the absorption rate; the farther away from this value, the smaller the absorption rate, the larger the bandwidth, and the smaller the corresponding Q-factor.

Figure 7f shows the variation curve of the absorption peak with *w*_2_. It should be noted that, limited by the size, the maximum value of *w*_2_ can only be 15 μm, at which point the resonant rings between adjacent units are actually connected, which will weaken the absorption peak caused by the coupling between adjacent units. This can be confirmed from the figure. When the value is small, the absorption peak is divided into two parts: one part is the absorption concentrated at low frequency, which is characterized by a decrease as the value increases. The other part is the high-frequency absorption, which increases with the value and moves toward a low frequency. From the curve trend and shape, it can be determined that the final absorption peak used for detection at 15 μm is actually the initial high-frequency absorption, while the low-frequency absorption is completely suppressed as the ring width increases, making the gap between adjacent units’ resonant rings gradually smaller until connected.

As mentioned above, the width of the gap on the bottom plate *w*_3_ does not affect the absorption. This judgment is made considering that the value of *w*_4_ is 20–40 μm, and that, without changing the shape of the gap, the width of *w*_3_ should not exceed 20 μm. This is because if the width of *w*_3_ is too large, the geometric structure essentially changes, and the gap exists only to cut off the current between the units rather than affect the current in the middle of the resonant unit.

**Figure 8:**
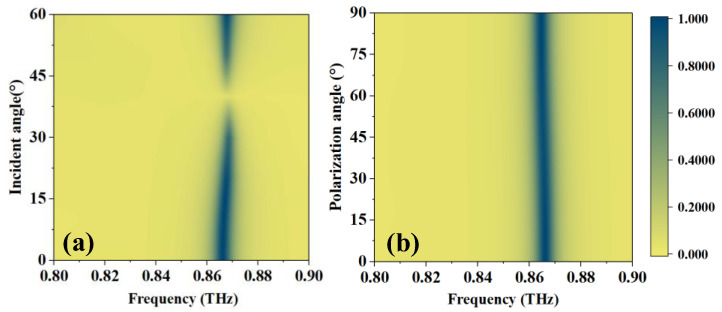
Absorptivity at different (**a**) *θ* and (**b**) *φ*.

The performance at different incidence angles *θ* and polarization angles *φ* is given in Figure 8. There is a minimal value near 40° as *θ* changes, and then it recovers. For *φ*, the absorption peak is stable, so the polarization is not sensitive. This feature allows for a wide range of signal source requirements for practical use. 

**Figure 9:**
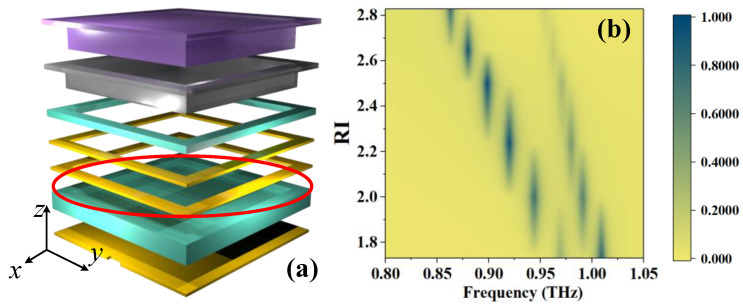
(**a**) Schematic diagram of double ring structure and (**b**) its performance.

As demonstrated in Figure 9a, a resonant ring of the same size is added at a position of 10 μm below the original resonant ring, which may excite new resonant peaks and provide absorption points with larger Q-factors. However, it may also produce redundant resonant peaks that cannot be suppressed. The absorption peak of the double-resonant ring structure in the initial state is shown in Figure 9b. Unfortunately, such a structure did not excite a steeper peak but destroyed the original resonance, causing the absorption rate to decrease overall, and it excited two absorption peaks with very close absorption peak values at RI = 2, further deteriorating the sensing effect, as displayed in Figure 9b. Therefore, for the existing structure, considering the simplicity of structure fabrication and effect, the single-resonant ring structure shows the best effect. Such a conclusion provides guidance for the optimization of sensing effects, that is, if further optimization of the Q-factor is desired, it can be optimized from the perspective of a planar structure and resonance principle, or similar. At least in terms of optimization complexity, vertical periodic structures are not preferred options. 

Table 4 lists the comparison with other studies, and it can be found that although this work is not outstanding in terms of performance, the characteristics of liquid crystal tuning in the same field are not common.

## 5. Conclusions

In summary, this paper proposes a liquid crystal-controlled metastructure sensor. Absorption can be caused by the resonance between adjacent resonant rings, and the absorption frequency point changes with the refractive index of the filling liquid. Sensing can be realized according to this phenomenon. With the help of liquid crystals, the detection range of the refractive index can be adjusted at different voltages. The device can be applied to the detection of biological solutions, such as creatinine concentrations in blood.

In the initial state, the detection range is 1.414–2.828, the frequency range of the absorption peak is 0.8485–1.028 THz, and the sensitivity is 123.8 GHz/RIU. When a voltage is applied to rotate the liquid crystal until it is fully biased, the detection range becomes 2.121–3.464, the frequency range is 0.7295–0.8328 THz, and the sensitivity is 75.6 GHz/RIU. The detailed performances are listed in Table 5. The refractive index of blood at different creatinine concentrations is usually between 2.5 and 2.7, which indicates that the existing detection range can meet the detection needs well and adapt to more application scenarios.

In addition, this paper analyzes the cause of absorption through the current and energy density diagrams and proposes a method to suppress the additional absorption peak, which can avoid absorption at the same frequency point under different refractive indices. 

Finally, the parameters of the device are discussed, including the effects of errors in the manufacturing process and the thickness errors of the sample liquid in actual use. The device is extremely stable at different angles; the incidence angle has a stable range of about 40°, and the polarization angle will not affect the performance. Furthermore, the number of resonant rings is also discussed, and it is clear that the optimal absorption can only be obtained at a single-resonant ring, and excess resonant rings will produce excess absorption peaks, which provides a guiding idea for the optimization of the structure.

This metastructure sensor has the advantages of real-time detection and a large detection range and can be used for biosensing, including, but not limited to, creatinine concentration measurements, providing new ideas for the design of tunable sensors.

## Figures and Tables

**Table 1:** The performance of the MS.

		Range	Sensitivity
Initial state	RI	1.414–2.828	123.8 GHz/RIU
	Frequency	0.8485–1.028 THz	
Full-bias state	RI	2.121–3.464	75.6 GHz/RIU
	Frequency	0.7295–0.8328 THz	

**Table 2:** Values of the parameters.

*L*_1_ (μm)	*w*_1_ (μm)	*w*_2_ (μm)	*t*_1_ (μm)
200	3	15	5
*t*_2_ (μm)	*t*_3_ (μm)	*t*_4_ (μm)	*t*_5_ (μm)
5	7	3	20
*t*_6_ (μm)		*t*_7_ (μm)	
3		20	

**Table 3:** RI of blood at different creatinine concentrations.

Creatinine Blood Sample Concentration (μmol L^−1^)	RI
80.9	2.661
81.43	2.655
82.3	2.639
83.3	2.610
84.07	2.589
85.28	2.565

**Table 4:** Comparison with other studies.

Ref.	Function	Tunable	Sensitivity	FOM	Liquid Crystal
[24]	Agricultural sensing	No	220.7 GHz/RIU	1.52	No
[26]	Thickness detection	No	6.61 GHz/mm	21.975	No
[29]	Biosensing	No	264 or 968 GHz/RIU	9.46	No
[33]	Biosensing	Yes	136.4–306.25 nm/RIU	1.5–10.3	No
This work	Biosensing	Yes	123.8 or 75.6 GHz/RIU	20 or 13	Yes

**Table 5:** Detailed performance of the device.

		Range	Sensitivity	FOM
Initial state	RI	1.414–2.828	123.8 GHz/RIU	20 RIU^−1^
	Frequency	0.8485–1.028 THz		
Full bias state	RI	2.121–3.464	75.6 GHz/RIU	13 RIU^−1^
	Frequency	0.7295–0.8328 THz		

## Data Availability

Samples of the compounds are available from the authors.
